# Antimalarial activity of 80 % methanolic extract of *Brassica nigra* (L.) Koch. (*Brassicaceae*) seeds against *Plasmodium berghei* infection in mice

**DOI:** 10.1186/s12906-015-0893-z

**Published:** 2015-10-15

**Authors:** Abrham Belachew Muluye, Eshetie Melese, Getnet Mequanint Adinew

**Affiliations:** Department of Pharmacy, Wollega University, Nekemte, Ethiopia; Department of Pharmacology, College of Medicine and Health Sciences, University of Gondar, Gondar, Ethiopia

**Keywords:** Antimalarial activity, *Brassica nigra*, Malaria, Mice, *Plasmodium berghei*

## Abstract

**Background:**

Resistances to currently available drugs and insecticides, significant drug toxicities and costs and lack of vaccines currently complicated the treatment of malaria. A continued search for safe, effective and affordable plant-based antimalarial agents thus becomes crucial and vital in the face of these difficulties. The aim of the study was to evaluate the antimalarial activity of 80 % methanolic extract of the seeds of *Brassica nigra* against *Plasmodium berghei* infection in mice.

**Method:**

Chloroquine sensitive *Plasmodium berghei* (ANKA strain) was used to test the antimalarial activity of the extract. In suppressive and prophylactic models, Swiss albino male mice were randomly grouped into five groups of five mice each. Group I mice were treated with the vehicle, group II, III and IV were treated with 100, 200, and 400 mg/kg of the extract, respectively and the last group (V) mice were treated with chloroquine (10 mg/kg). The level of parasitemia, survival time and variation in weight of mice were used to determine the antimalarial activity of the extract.

**Results:**

Chemosuppressive activities produced by the extract of the seeds of *Brassica nigra* were 21.88, 50.00 (*P* < 0.01) and 53.13 % (*P* < 0.01), while the chemoprophylactic activities were 17.42, 21.21 and 53.79 % (*P* < 0.05) at 100, 200 and 400 mg/kg of the extract, respectively as compared to the negative control. Mice treated with 200 and 400 mg/kg extract were significantly (*P* < 0.05) lived longer and gained weight as compared to negative control in 4-day suppressive test.

**Conclusion:**

From this study, it can be concluded that the seed extract of *Brassica nigra* showed good chemosuppressive and moderate chemoprophylactic activities and the plant may contain biologically active principles which are relevant in the treatment and prophylaxis of malaria, thus supporting further studies of the plant for its active components.

## Background

Malaria is a serious hazard to humanity and the major cause of mortality and morbidity in the malaria-endemic countries. Even though the distribution of the disease is substantially varied, sub-Saharan Africa, Asia and Central and Latin America are the most affected regions. About 50 % of populations in the world live in malaria risk areas. In 2013, it was estimated that 198 million cases of malaria and 584,000 malarial deaths have been occurred in the world. The burden of the disease is heaviest in Africa, where 82 and 90 % of all global cases and deaths were occurred, respectively [1].

Resistances of mosquitoes to insecticides and parasites to antimalarial drugs especially to the current most effective and newest artemisinin derivatives have led to an increase in severe malaria and complicate the eradication of the disease as well as the resurgence of malaria [2]. While there is much need and there is currently no alternative to the precious artemisinin derivatives, the antimalarial drug development pipeline remains sadly thin with little chemical diversity [3]. Preventive and treatment strategies of malaria are not only continuously hampered by the issues of resistance but also considerable costs and logistical problems especially in poor malarious countries as well as a challenge of having effective vaccines create a great and urgent concern for the treatment of the disease [4].

Additionally, many antimalarial drugs in use today have high toxicity and low therapeutic margin of indices that exposes patients’ additional harm and health expenditure [5]. The efficacy, safety and quality of most medicinal plant products in traditional medicine are not validated and standardized. Medicinal plants used in traditional medicine should, therefore, be studied for their safety and efficacy and their quality as well to safe guard the health of customers [6].

In the face of current resistance problems, medicinal plants are a potential source of new, effective and affordable antimalarial agents. However, most of these plants, represent a virtually unlimited reservoir of molecules, are not explored chemically and can constitute lead molecules for development of new antimalarial drugs. Therefore, antimalarial drug discovery from such plants is currently more targeted because histories proved that plants are richest sources of antimalarial phytochemicals [7, 8].

Quinine and artemisinin, two main classes of antimalarials, currently in use have been provided by *Cinchona* species and *Artemisia annua* traditionally used for malaria for a long period of times, respectively. Therefore, medicinal plants have made and continue to make a great contribution to antimalarial chemotherapy as they contain molecules with a great variety of structures and biological activities [9, 10].

The genus *Brassica* is reported to comprise over 150 species, of which five including *Brassica nigra* are found in Ethiopian flora. *Brassica nigra* (local Ethiopian Amharic name: *Senafitch*) is an annual, erect, aromatic, up to 100 cm tall with freely and widely branching, and glabrate weedy herb [11]. *Brassica nigra* is used throughout the world for various aspects such as food, vegetable, medicinal and industrial products. The medicinal effect is mainly confined to its seeds. The seeds have been used as tonic, digestive, emetic, appetite stimulant, laxatives, antiseptics and for carcinoma and tumors [12], liver and spleen indurations, abscesses, colds, headaches, rheumatism, edema, neuralgia, alopecia, epilepsy, snakebite, toothache, hiccup, pneumonia [13] and malaria [14, 15]. Its seeds are powdered and a paste have been made with water and then eaten with and without “*Injera*” (a flat Ethiopian bread resembling a large pancake) for the prevention and treatment of malaria, respectively, in Ethiopia [15].

The seed extract of *Brassica nigra* showed potent antiepileptic activity [16] which might be important for the management of cerebral malaria which is associated with protracted and/or febrile convulsions. The plant is also endowed with various pharmacologically active phytochemicals, such as alkaloids, flavonoids, tannins, terpenoids, and others [17, 18], with reported antiplasmodial activity [10] and hence this also justified the current study.

Considering all the above since there is no previous report, laboratory-based evidence for its antimalarial effectiveness is found essential. This study is, therefore, designed to evaluate the antimalarial activity of the 80 % methanolic extract of the seeds of *Brassica nigra* against *Plasmodium berghei* infection in mice.

## Methods

### Collection and preparation of the plant materials

The fresh seeds of *Brassica nigra* were collected from the farmers’ farmland in November 2014, around Gondar town, 728 km from Addis Ababa, Northwest Ethiopia. The plant was identified and authenticated and a voucher specimen (with a voucher number of AB05) was deposited at National Herbarium, Addis Ababa University, Ethiopia. The seeds of *Brassica nigra* were cleaned and air-dried in the shade at room temperature. The dried seeds were coarsely powdered using mortar and pestle. Then the powdered plant materials were stored in a plastic container and they were kept at room temperature until extraction.

### Extraction of the plant materials

The powdered samples of seeds of *B. nigra* (325 g) were weighed by sensitive balance. They were extracted by maceration with 80 % methanol (1:10 ratio of powdered plant material (in gram) and solvent (in millitre), respectively) for three consecutive days with occasional stirring. After 72 h, the mixture was filtered with Whatman filter paper no 1 (Whatman, England). The residue was re-macerated twice for the same duration of hours and then the mixture were filtered [19]. The combined filtrates were dried by oven (250 V, France) set below 40 °C. The weight of the dried extract was measured to determine the percentage yield. Finally, the dried extract was then transferred into a vial and it was kept in a refrigerator until further use.

### Experimental animals

Fifty Swiss albino male mice, weighing 25 to 32 g and 6 to 8 weeks old inbred at the Animal House of the Department of Pharmacology, University of Gondar were used. They were housed in plastic cages with softwood shavings and chips as beddings. They were exposed to a 12:12 dark-to-light cycle. They had free access to pellet diet and clean drinking water. All mice were acclimatized to the working environment one week before the beginning of the experiment [20].

### Parasites

Chloroquine sensitive *Plasmodium berghei* (ANKA strain) was used for induction of malaria in experimental mice. Mice previously infected with *P. berghei* were used as donor. The donor *P. berghei* infected mice were obtained from Aklilu Lemma Institute of Pathobiology, Addis Ababa University, Ethiopia. The parasites were subsequently maintained in the laboratory by serial passage of blood from donor infected mice to naive one via intra-peritoneal (IP) route on weekly basis [21].

### Drugs and reagents

Chloroquine (Addis Pharmaceuticals Factory, Ethiopia), methanol (Okhla Industrial, India), Giemsa (Sciencelab, USA), trisodium citrate (Deluxe Scientific surgico, India), ketamine (Rotexmedica, Germany) and normal saline (Addis Pharmaceuticals Factory, Ethiopia) were used. All reagents were analytically graded and procured from certified suppliers.

### Study design and sampling method

Experimental study design was used. Simple random sampling technique was employed for grouping of experimental animals and assignments of treatments.

### Inoculum preparation

The parasitemia of the previously *P. berghei* infected donor mouse were determined. After anaesthetizing with ketamine, the blood from these mice was collected via cardiac puncture with a rising parasitaemia of 30 to 35 % into a test tube having 0.5 % trisodium citrate which was added as anticoagulant. The blood was then diluted with normal saline (0.9 %) to give 2 × 10^7^ IRBCs in an injection volume of 0.2 ml. The amount of normal saline used in the dilution was determined by the level of parasitaemia (%) of the infected donor mice. If 0.5 ml of blood was removed then dilution was done with (X-0.5) ml of saline, where X was the % parasitaemia. Each mouse was then infected by injecting 0.2 ml of this diluted blood via IP which contained 2 × 10^7^ IRBCs, which produces a steadily rising infection in mice [21, 22].

### Grouping and dosing of animals

For each model (suppressive and prophylactic), twenty five mice were grouped into five groups of five mice each. Group I mice were treated with the vehicle (distilled water, 10 ml/kg, served as negative control), group II, III and IV mice were treated with 100, 200, and 400 mg/kg of crude extract, respectively and the last group (V) mice were treated with the standard drug (chloroquine, 10 mg/kg, served as positive control).

The safety data of the plant was obtained from Praveen et al. [16] as the crude methanolic extract of the seeds of *B. nigra* was safe at 2000 mg/kg in mice and this was a basis for the selection of the three dose levels (100, 200 and 400 mg/kg) of the extract for the current antimalarial activity evaluation. As people traditionally use the plant material for treatment and prevention of malaria orally [14], each treatment was administered through oral route using oral gavage to ensure safe ingestion of the preparations.

### Four-day suppressive antimalarial test

*Plasmodium berghei* four-day suppressive test, the most widely used preliminary test, in which the efficacy of four daily doses of new compounds is measured by comparison of blood parasitaemia and survival times of treated and untreated mice. The test provides a preclinical indication of potential bioactivity of the extract [21, 23]. The test was done by a method described by Fidock et al. [21]. After standard parasite inoculation (2 × 10^7^*P. berghei* IRBCs, 0.2 ml), 25 mice were randomly divided into five groups with five mice each as above mentioned in grouping of animals. Treatment was started three hours after infection and then continued for three consecutive days (i.e., from D_0_ to D_3_). On the 5th day (D_4_), thin blood films were made from the tail of each mouse on a microscopic slide (Citoplus, China).

### Prophylactic antimalarial test

Compounds identified as being active in four-day suppressive assays can subsequently be further examined through the use of several secondary tests in mice from which prophylactic test are one of them. Evaluation of prophylactic potential of the extract was done according to Fidock et al. [21]. Twenty five mice were randomly grouped into five groups as mentioned above accordingly and then treated for four consecutive days (D_0_ to D_3_). On the fifth day (D_4_), a standard inoculum (2 × 10^7^*P. berghei* IRBCs, 0.2 ml) was administered by IP to each mouse. After 72 h of infection (D_7_), thin blood smears were prepared from tail of each mouse on a microscopic slide.

### Peripheral blood smear preparation

For both models (suppressive and prophylactic), thin smears of blood were made from the tail of each mouse on the fifth day (D_4_) and eighth day (D_7_), respectively. The smears were applied on microscopic slides and the bloods were drawn evenly across a second slide to make thin blood films and allowed to dry at room temperature. Then they were fixed with 100 % methanol and stained with 10 % Giemsa stain at pH 7.2 for 15 min.

### Parasitemia determination

Each stained slide for each mouse was examined under microscope (Olympus, Japan). The parasitaemia was determined by counting the number of parasitized erythrocytes in random fields of the microscope. The smears were counted by a laboratory technician to make the reader blind to the category. Percent parasitaemia and percent suppression were calculated by using the following formula, respectively [21, 24].

$$ \%\ \mathrm{Parasitaemia} = \frac{\mathrm{Number}\ \mathrm{of}\ \mathrm{infected}\ \mathrm{RBCs}}{\mathrm{Total}\ \mathrm{Number}\ \mathrm{of}\ \mathrm{RBCs}}\times 100 $$

$$ \%\ \mathrm{Suppression} = \frac{\left(\%\ \mathrm{Parasitemia}\ \mathrm{of}\ \mathrm{Negative}\ \mathrm{Control}\hbox{--} \%\ \mathrm{Parasitemia}\ \mathrm{of}\ \mathrm{Treated}\ \mathrm{Group}\right)}{\%\ \mathrm{Parasitemia}\ \mathrm{of}\ \mathrm{Negative}\ \mathrm{Control}}\times 100 $$

### Determination of mean survival time

Mean survival time (MST) is another parameter that is commonly used to evaluate the efficacy of antimalarial plant extracts [25]. An extract that results in survival time greater than that of infected non-treated mice was considered as active. Mortality was monitored daily and the number of the days from the time of infection up to death was recorded for each mouse in the treatment and control groups throughout the follow-up period and the MST was calculated for each group by using the following formula.

$$ \mathrm{M}\mathrm{S}\mathrm{T} = \frac{\mathrm{Sum}\ \mathrm{of}\ \mathrm{S}\mathrm{urvival}\ \mathrm{T}\mathrm{ime}\ \mathrm{of}\ \mathrm{All}\ \mathrm{Mice}\ \mathrm{in}\ \mathrm{a}\ \mathrm{Group}\ \left(\mathrm{days}\right)}{\mathrm{Total}\ \mathrm{Number}\ \mathrm{of}\ \mathrm{mice}\ \mathrm{in}\ \mathrm{T}\mathrm{hat}\ \mathrm{Group}} $$

### Body weight determination

Body weight loss is one feature of rodent malaria infections. Body weight change is another parameter used to assess the effectiveness of plant extracts [24]. The body weight of each mouse in all groups was taken before infection (D_0_) and treatment (D_1_) and after treatment (D_4_) and infection (D_7_), in suppressive and prophylactic tests, respectively. The weight of each mouse was measured using sensitive electrical balance (ADP 720 L, Adam Equipment). Then, % body weight change of extract treated groups was compared with the control groups.

### Data analysis

The data of the study were expressed as mean ± SEM (standard error of mean) for each group of experiments. Data on the levels of parasitemia, variations in body weight and survival times were analyzed using windows SPSS version 16.0. The differences between means of measured parameters were compared using one-way ANOVA followed by Turkey’s HSD multiple comparison test and paired sample t-test (two-tailed). The *P* values < 0.05 were regarded as statistically significant.

### Data quality control

The quality of data was assured by using randomization during grouping of experimental animals and assignments of treatments. Codes were used for all microscopic slides. Parasitized RBCs were counted blindly by a laboratory technician.

### Ethical consideration

During experimental procedures, experimental animals were handled and cared according to the internationally accepted laboratory animals’ use, care and welfare guideline [20]. Ethical clearance was requested to and obtained from animal ethics committee of Department of Pharmacology, School of Pharmacy, University of Gondar.

## Results

### Percentage yield of the plant material

The percentage yield of the dry matter of the 80 % methanolic crude extract of the seeds of *Brassica nigra* was 9.85 % w/w. Its actual yield was 32 g. It was red in its colour. Hygroscopic powder was formed after drying.

### Four-day suppressive antimalarial activity of the extract

The methanolic crude extract of the seeds of *Brassica nigra* showed chemosuppressive activity against *Plasmodium berghei* infection in mice with 21.88, 50.00 and 53.13 % chemosuppressions at 100, 200 and 400 mg/kg of the extract, respectively, shown in Table 1.

**Table 1:** Four-day chemosuppressive activity of methanolic crude extract of the seeds of *Brassica nigra* against *Plasmodium berghei* infection in mice

Treatments	Doses	% Parasitemia	% Chemosuppression
Distilled water	10 ml/kg	25.60 ± 2.40	0.00
*Brassica nigra* seed extract	100 mg/kg	20.00 ± 2.53	21.88^b3^
	200 mg/kg	12.80 ± 0.80	50.00^a2,b2^
	400 mg/kg	12.00 ± 2.83	53.13^a2,b1^
Chloroquine	10 mg/kg	1.40 ± 0.75	94.53^a3^

*Values are expressed as Mean ± SEM; n = 5. Where a = as compared to negative control; b = as compared to positive control; 1 = P < 0.05; 2 = P < 0.01 and 3 = P < 0.001*

Even though there were differences in parasitemia suppression among extract treated groups, they were statistically non-significant.

### Chemoprophylactic antimalarial activity of the extract

The extract of *Brassica nigra* also showed chemoprophylactic activity against *Plasmodium berghei* infection in mice with 17.42, 21.21 and 53.79 % suppressions at 100, 200 and 400 mg/kg of the extract, respectively, shown in Table 2.

**Table 2:** Chemoprophylactic activity of methanolic crude extract of the seeds of *Brassica nigra* against *Plasmodium berghei* infection in mice

Treatments	Doses	% Parasitemia	% Chemosuppression
Distilled water	10 ml/kg	26.40 ± 5.80	0.00
*Brassica nigra* seed extract	100 mg/kg	21.80 ± 2.65	17.42^b2^
	200 mg/kg	20.80 ± 3.01	21.21^b2^
	400 mg/kg	12.20 ± 1.36	53.79^a1^
Chloroquine	10 mg/kg	1.00 ± 0.45	96.21^a3^

*Values are expressed as Mean ± SEM; n = 5. Where a = as compared to negative control; b = as compared to positive control; 1 = P < 0.05; 2 = P < 0.01 and 3 = P < 0.001*

There were no statistically significant chemoprophylactic differences among extract treated groups as that of four-day suppressive test.

### Effect of extract on survival times of mice

In the four-day suppressive test, 200 and 400 mg/kg extract treated groups lived longer than the corresponding negative control. There were no significant differences in survival times among extract treated groups. In prophylactic test, however, the survival times did not prolonged significantly as compared to negative control, shown in Table 3.

**Table 3:** Effect of crude methanolic extract of the seeds of *B. nigra* on the mean survival times of *P. berghei* infected mice in both suppressive and prophylactic tests

Tests	Treatments	Doses	Mean Survival Times (Days)
Suppressive	Distilled water	10 ml/kg	7.20 ± 0.37
	*Brassica nigra* seed extract	100 mg/kg	7.60 ± 0.25^b2^
		200 mg/kg	9.00 ± 0.45^a1,b2^
		400 mg/kg	9.00 ± 0.71^a1,b2^
	Chloroquine	10 mg/kg	15.00 ± 0.00^a2^
Prophylactic	Distilled water	10 ml/kg	6.80 ± 0.20
	*Brassica nigra* seed extract	100 mg/kg	7.00 ± 0.32^b2^
		200 mg/kg	7.60 ± 0.25^b2^
		400 mg/kg	7.80 ± 0.37^b2^
	Chloroquine	10 mg/kg	14.60 ± 0.40^a2^

*Values are expressed as Mean ± SEM; n = 5. Where a = as compared to negative control; b = as compared to positive control; 1 = P < 0.05 and 2 = P < 0.001*

### Effect of extract on body weights of mice

In the 4-day suppressive test, all extract treated groups prevented body weight loss as compared to negative control. However, there were no significant differences in weight gain among extract treated groups, shown in Table 4.

**Table 4:** Effect of crude methanolic extract of the seeds of *B. nigra* on the body weights of *P. berghei* infected mice in suppressive test

Treatments	Doses	Body Weight (gm)		
		D_0_-Weight	D_4_-Weight	Wt Change (%)
Distilled water	10 ml/kg	29.64 ± 0.34	29.30 ± 0.32	−1.15
*Brassica nigra* seed extract	100 mg/kg	30.38 ± 1.89	30.48 ± 2.05	0.33
	200 mg/kg	26.68 ± 1.57	26.92 ± 1.57	0.90^a1^
	400 mg/kg	29.26 ± 1.41	29.62 ± 1.42	1.23^a1^
Chloroquine	10 mg/kg	28.18 ± 1.64	28.58 ± 1.65	1.42^a2^

*Values are expressed as Mean ± SEM; n = 5. Where a = as compared to negative control; 1 = P < 0.01 and 2 = P < 0.001*

In the prophylactic test, however, there were non-significant body weight gains in extract treated groups as compared to negative control, shown in Table 5.

**Table 5:** Effect of crude methanolic extract of the seeds of *B. nigra* on the body weights of *P. berghei* infected mice in prophylactic test

Treatments	Doses	Body Weight (gm)		
		D_1_-Weight	D_7_-Weight	Wt Change (%)
Distilled water	10 ml/kg	24.94 ± 1.99	24.48 ± 2.00	−1.84
*Brassica nigra* seed extract	100 mg/kg	26.80 ± 0.99	27.00 ± 0.94	0.75
	200 mg/kg	25.84 ± 2.42	26.06 ± 2.50	0.85
	400 mg/kg	28.10 ± 1.56	28.42 ± 1.68	1.14
Chloroquine	10 mg/kg	26.86 ± 1.13	27.34 ± 1.17	1.79^a1^

*Values are expressed as Mean ± SEM; n = 5. Where a = as compared to negative control and 1 = P < 0.05*

## Discussion

The antimalarial activity of the crude methanolic extract of the seeds of *Brassica nigra,* used in traditional medicine in Ethiopia and elsewhere, against *Plasmodium berghei* infection in mice in both four-day suppressive and prophylactic test models are reported.

The percentage suppression of parasitaemia of the extract treated groups changed significantly from those in the negative control group showing that the extract has antimalarial activity supporting the folk use of the plant as antimalarial herb. A compound is considered as active when percent suppression in parasitemia is 30 % or more [26, 27], which supports the findings of the current study.

The mice treated with 200 and 400 mg/kg of extract showed significant 50.00 and 53.13 % chemosuppressions, respectively, as compared to negative control. The chemosuppressive effects of the extract of the seeds of *Brassica nigra* in the current study were in agreement with other reports on medicinal plants used for malaria such as *Echinops kebericho* [28]; *Agelanthus dodeneifolius* [29]; *Sida rhombifolia* [30] and *Phytolacca dodecandra* [31] which showed significant 29.46 % and 57.29; 48.02 and 50.50; 50.10 and 52.30; and 50.93 and 55.24 % chemosuppressions, respectively, with similar doses.

According to Deharo et al. [32], in vivo antimalarial activity of plant extracts can be categorized as: moderate, good and very good if the extract showed 50 % or more chemosuppression at 500, 250 and 100 mg/kg/day extract dose, respectively. Hence, the current finding revealed that hydroalcoholic seed extract of *B. nigra* has good 4-day suppressive antimalarial activity.

The methanolic seed extract of *B. nigra* also showed significant chemoprophylactic activity against residual infection at 400 mg/kg as 53.79 % chemosuppression. The current finding was in agreement with other reports on medicinal plants used for malaria such as *Languas galangal* [33] and *Agelanthus dodeneifolius* [29] which showed significant 52.00 and 56.00 % chemosuppression, respectively. According to Deharo et al. [32] in vivo antimalarial activity classification, the methanolic crude extract of the seeds of *Brassica nigra* showed moderate chemoprophylactic antimalarial activity.

In both models (suppressive and prophylactic), chloroquine, the current antimalarial drug, was used as a reference drug [21, 25] and it showed parasitemia suppression near to undetectable levels of 94.53 and 96.21 %, respectively. It leads to decreased parasitemia and resultant recovery of severe malaria by reducing parasite nutrient intake as well as by interfering with parasite metabolic machinery involved with iron [33]. The chemosuppression showed by chloroquine in the current study was in agreement with other studies on medicinal plants used for malaria such as *Artemisia annua* [34], *Annona senegalensis* [35] and *Adhathoda schimperiana* [36] which showed 90.48, 96.20 and 97.80 %, respectively.

The pharmacological (i.e., the antiplasmodial) activities of plants are due to the presence of bioactive secondary metabolites in the crude plant material [37]. Basically, different secondary metabolites, such as alkaloids, glycosides, phenols, terpenoids and sterols [17], flavonoids and tannins [18], have been reported from the seed extract of *Brassica nigra*. These metabolites have antiplasmodial activities [9, 10, 37, 38]. However, the active compound(s) responsible for this observed antiplasmodial activity need to be identified.

Therefore, the antiplasmodial activity observed in this plant could have resulted from single or in combined action of the above metabolites [39]. The possible mechanisms of antiplasmodial activity might be through anti-oxidation and free radical scavenging [21], immunomodulatory [35], intercalation in DNA, inhibition of protein synthesis, interference with the invasion of new RBCs by parasites, or by any other unknown mechanisms [29, 39].

Mean survival time is another parameter evaluates the antimalarial activity of plant extracts. Accordingly, a plant material that can prolong the survival time of infected experimental animals compared to the negative controls are considered as active agents against malaria [25]. An extract that results in survival time greater than that of infected non-treated mice was considered as active [27]. In this study the mice treated with 200 and 400 mg of extract had significantly lived longer than negative control in four-day suppressive test. This might be due to the antiplasmodial activity of the extract. However, the survival times of mice treated with the extract were shorter as compared to positive control; this might be the fast elimination phase of the extract. The current findings were in agreement with studies done on medicinal plants used for malaria such as *Dodonaea angustifolia* [40] and *Nigella sativa* [41].

Body weight loss is one manifestation of *Plasmodium berghei*-infected mice. This is due to the depressant action on the appetite of the mice and the consequences of disturbed metabolic function and hypoglycemic effect of the parasite [22]. Body weight change of mice is also another parameter that evaluates the antimalarial activity of plant extracts [42]. Mice treated with 200 and 400 mg/kg of extract showed a significant increased in body weight gain as compared to negative control, hence, this might tell us the antiplasmodial activity of the extract.

The plant possesses other pharmacologic benefits which might in part lead to increased weight gain including appetite stimulant activity [12] and it is nutritionally endowed with vitamin Bs such as vitamin B_1_, thiamine which maintains appetite and growth; vitamin B_2_, riboflavin which prevents skin lesions and weight loss; and vitamin B_3_, niacin which maintains the normal function of the nervous system and the gastrointestinal tract [13]. However, the weight gains of extract treated mice were insignificant as compared to the negative control in prophylactic test. This might be the rapid hepatic clearance of the active component of the plant, which was given before infection at which the basal metabolic rate of the mice is active, involved in weight gain. This finding was in agreement with other study on medicinal plant, *Faidherbia albida* [43], used for malaria.

## Conclusion

From this study, it can be concluded that the seeds of *Brassica nigra* showed good chemosuppressive and moderate chemoprophylactic antimalarial activities against *Plasmodium berghei* infection in mice. Accordingly, with the essence of further studies this plant could serve as the potential source of new and novel antimalarial leads and/or drugs for the treatment and prevention of malaria.
